# Admission hyperglycaemia as a predictor of mortality in patients hospitalized with COVID-19 regardless of diabetes status: data from the Spanish SEMI-COVID-19 Registry

**DOI:** 10.1080/07853890.2020.1836566

**Published:** 2020-11-04

**Authors:** Francisco Javier Carrasco-Sánchez, Mª Dolores López-Carmona, Francisco Javier Martínez-Marcos, Luis M. Pérez-Belmonte, Alicia Hidalgo-Jiménez, Verónica Buonaiuto, Carmen Suárez Fernández, Santiago Jesús Freire Castro, Davide Luordo, Paula Maria Pesqueira Fontan, Julio César Blázquez Encinar, Jeffrey Oskar Magallanes Gamboa, Andrés de la Peña Fernández, José David Torres Peña, Joaquim Fernández Solà, Jose Javier Napal Lecumberri, Francisco Amorós Martínez, María Esther Guisado Espartero, Carlos Jorge Ripper, Raquel Gómez Méndez, Natalia Vicente López, Berta Román Bernal, María Gloria Rojano Rivero, José Manuel Ramos Rincón, Ricardo Gómez Huelgas, for the SEMI-COVID-19 Network

**Affiliations:** a Internal Medicine Department, Juan Ramón Jiménez University Hospital, Huelva, Spain; b Internal Medicine Department, Málaga Regional University Hospital, Málaga, Spain; c Clinical Infectious Disease Unit, Juan Ramón Jiménez University Hospital, Huelva, Spain; d Internal Medicine Department, La Princesa University Hospital, Madrid, Spain; e Internal Medicine Department, A Coruña University Hospital, A Coruña, Spain; f Internal Medicine Department, Infanta Cristina University Hospital, Parla, Spain; g Internal Medicine Department, Santiago Clinical Hospital, Santiago de Compostela, Santiago, Spain; h Internal Medicine Department, Torrevieja University Hospital, Torrevieja, Spain; i Internal Medicine Department, Nuestra Señora del Prado Hospital, Talavera de la Reina, Spain; j Internal Medicine Department, Son Llàtzer University Hospital, Palma de Mallorca, Spain; k Lipis and Atherosclerosis Unit, Department of Interna Medicine, Maimonides Biomedical Research Institute of Córdoba (IMIBIC), Reina Sofia University Hospital, University of Córdoba, Spain; l CIBER Fisiopatologia de la Obesidad y Nutrición (CIBEROBN), Insituto de Salud Carlos III, Córdoba, Spain; m Internal Medicine Department, Barcelona Clinical Hospital, Barcelona, Spain; n Internal Medicine Department, Marqués de Valdecilla University Hospital, Santander, Spain; o Internal Medicine Department, Vinalopó University Hospital, Elche, Spain; p Internal Medicine Department, Infanta Margarita Hospital, Cabra, Spain; q Internal Medicine Department, Insular de Gran Canaria Hospital, Las Palmas de Gran Canaria, Spain; r Internal Medicine Department, Lucus Augusti University Hospital, Lugo, Spain; s Internal Medicine Department, Sureste University Hospital, Arganda del Rey, Spain; t Internal Medicine Department, Doctor José Molina Orosa Hospital, Arrecife, Spain; u Internal Medicine Department, Infanta Elena Hospital, Huelva Hospital, Huelva, Spain; v Internal Medicine Department, Alicante General University Hospital, Alicante, Spain

**Keywords:** SARS-CoV-2, COVID-19, hyperglycaemia, mortality, diabetes

## Abstract

**Background:**

Hyperglycaemia has emerged as an important risk factor for death in coronavirus disease 2019 (COVID-19). The aim of this study was to evaluate the association between blood glucose (BG) levels and in-hospital mortality in non-critically patients hospitalized with COVID-19.

**Methods:**

This is a retrospective multi-centre study involving patients hospitalized in Spain. Patients were categorized into three groups according to admission BG levels: <140 mg/dL, 140–180 mg/dL and >180 mg/dL. The primary endpoint was all-cause in-hospital mortality.

**Results:**

Of the 11,312 patients, only 2128 (18.9%) had diabetes and 2289 (20.4%) died during hospitalization. The in-hospital mortality rates were 15.7% (<140 mg/dL), 33.7% (140–180 mg) and 41.1% (>180 mg/dL), *p*<.001. The cumulative probability of mortality was significantly higher in patients with hyperglycaemia compared to patients with normoglycaemia (log rank, *p*<.001), independently of pre-existing diabetes. Hyperglycaemia (after adjusting for age, diabetes, hypertension and other confounding factors) was an independent risk factor of mortality (BG >180 mg/dL: HR 1.50; 95% confidence interval (CI): 1.31–1.73) (BG 140–180 mg/dL; HR 1.48; 95%CI: 1.29–1.70). Hyperglycaemia was also associated with requirement for mechanical ventilation, intensive care unit (ICU) admission and mortality.

**Conclusions:**

Admission hyperglycaemia is a strong predictor of all-cause mortality in non-critically hospitalized COVID-19 patients regardless of prior history of diabetes.

**KEY MESSAGE**
- Admission hyperglycaemia is a stronger and independent risk factor for mortality in COVID-19.
- Screening for hyperglycaemia, in patients without diabetes, and early treatment of hyperglycaemia should be mandatory in the management of patients hospitalized with COVID-19.
- Admission hyperglycaemia should not be overlooked in all patients regardless prior history of diabetes.

## Introduction

Since the severe acute respiratory syndrome coronavirus 2 (SARS-CoV-2) pandemic emerged in China, more than 300,000 confirmed cases and approximately 28,500 coronavirus disease 2019 (COVID-19) related deaths have been reported in Spain as of the date of writing of this article [1].

Acute hyperglycaemia has been associated with in-hospital complications in non-critically ill patients with and without diabetes mellitus (DM) [2]. In regard to COVID-19, elevated blood glucose (BG) levels are also associated with in-hospital complications, including mechanical ventilation requirement, intensive care unit (ICU) admission and death [3–7]. Thus, hyperglycaemia, particularly upon admission, could be a marker of poor prognosis regardless of diabetes status. This suggests that elevated BG may play a decisive role in the severity of the disease at an early stage [8–10].

There are at least two plausible reasons why hyperglycaemia, particularly acute hyperglycaemia, could be harmful in patients with COVID-19 [11]. First, SARS-CoV-2 could infect endocrine pancreas cells through their expression of angiotensin-converting enzyme 2 (ACE2) receptors, resulting in an impairment in β-cell insulin secretion [12]. Second, inflammation during COVID-19 could also generate insulin resistance. Both mechanisms combined could induce hyperglycaemia in early stages of the disease. Once a patient presents with hyperglycaemia, it could play a direct role in worsening the infection. Hyperglycaemia upregulates ACE2 expression and induces glycosylation ofACE2, facilitating the invasion of cells by SARS-CoV-2 [13].

Although several studies and meta-analyses have shown that patients with diabetes have a significantly higher risk of severe COVID-19 and increased mortality rates [14–16], the impact of hyperglycaemia itself, rather than the presence of DM, has not been sufficiently described in non-critically patients hospitalized with COVID-19.

Therefore, our study sought to evaluate whether acute hyperglycaemia at admission, independently of diabetes status, was associated with all-cause in-hospital mortality in a large cohort of patients with SARS-CoV-2 infection in Spain. Additionally, we explored the relationship between high BG and length of stay (LOS), ICU admission and/or mechanical ventilation.

## Methods

### Study design and population

The SEMI-COVID-19 Registry is an ongoing nationwide, multicentre, observational, retrospective cohort registry involving 109 hospitals in Spain. It includes consecutive patients ≥ 18 years of age hospitalized from 1 March 2020 to 31 May 2020, who were admitted with COVID-19 confirmed microbiologically by reverse transcription polymerase chain reaction (RT-PCR) testing of a nasopharyngeal sample, sputum or bronchoalveolar lavage samples. Exclusion criteria were subsequent admissions of the same patient and denial or withdrawal of informed consent. Patients were treated at their attending physician’s discretion, according to local protocols and their clinical judgement.

### Registry information and data collection

Information on the registry is available in a previous published study, which includes all necessary details about procedures and describes the baseline characteristics of all patients included. Data are collected retrospectively and include approximately 300 variables grouped under various headings, such as epidemiological data, medical history and use of medications, symptoms and physical examination findings at admission, laboratory and diagnostic imaging tests, pharmacological treatment and so on [17]. Patients were classified as diabetic when diabetes was present in the history profile or they took antidiabetic drugs before admission. The management of hyperglycaemia during hospitalization was using insulin in basal bolus regimen according to local protocols.

The Spanish Agency of Medicines and Medical Devices (AEMPS, for its initials in Spanish) has ruled that due to the nature of the registry, the study only required the approval of Ethics Committees. The SEMI-COVID-19 Registry was approved by the Provincial Research Ethics Committee of Málaga (Spain). Informed consent was obtained from all patients. When it was not possible to obtain informed consent in writing due to biosafety concerns or if the patient had already been discharged, informed consent was requested verbally and noted on the medical record. The STROBE statement guidelines were followed in the conduct and reporting of the study.

### Study outcomes

The primary endpoint was all-cause mortality during hospitalization according to BG levels at admission. The secondary outcomes were LOS and the composite of the invasive or non-invasive mechanical ventilation, ICU admission or death. The follow-up period was from admission to discharge or death.

### Statistical analysis

BG levels obtained from a sample taken at admission were categorized into three groups according to standard glycaemic targets in hospitalized patients: <140 mg/dL, 140–180 mg/dL and >180 mg/dL. Hypoglycaemia was defined as a BG level below 70 mg/dL. Baseline characteristics were described according to these groups. Continuous variables were tested for normal distribution using Kolmogorov–Smirnov’s test.

Results are shown as means (standard deviation, SD) or medians (25th to 75th percentile) for continuous variables and numbers (%) for categorical variables.

To compare baseline demographic data and clinical characteristics among the BG level groups, we used analysis of variance (ANOVA) or the Kruskal–Wallis test for continuous variables. Differences in proportions were analysed using the chi-square test. Correlations between admission serum glucose levels and the main quantitative variables were calculated using Spearman's rank correlation coefficient test. In addition, the association between BG levels and death was analysed using Kaplan–Meier’s survival curves; the log-rank test was used to compare survival curves according to the BG level groups. A multivariate Cox proportional hazard regression model with BG levels as the predictor variable for all-cause mortality was used to estimate hazard ratios (HRs) with a 95% confidence interval (CI). Multivariate model was developed by using a forward stepwise method, including all variables with *p*<.1 on the univariate analysis. We also used a logistic regression to evaluate the relationship between BG levels and the composed secondary endpoint. All statistical analyses were performed using SPSS software (version 26.0, Chicago, IL). A two-sided *p* value <.05 was considered statistically significant.

## Results

### Baseline characteristics and correlation with blood glucose

**Figure 1.**
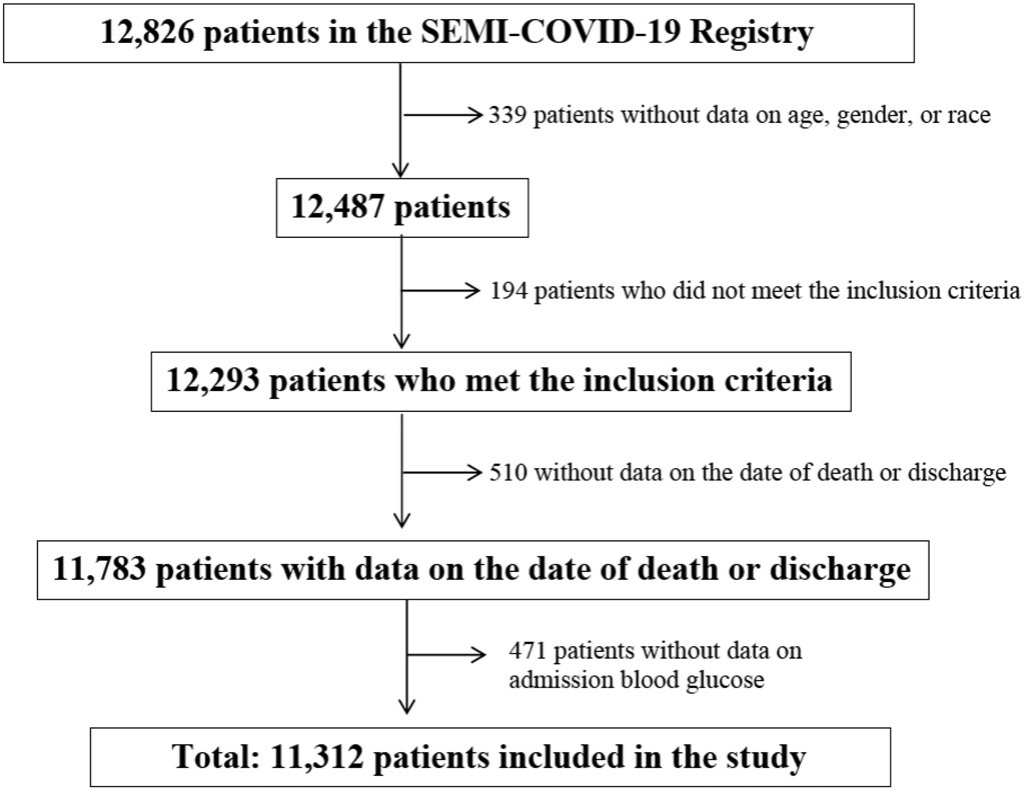
Patient inclusion flowchart.

Clinical characteristics were collected from a total of 11,312 participants out of the 12,826 confirmed cases of COVID-19 included in the registry as of 29 May 2020. A flowchart illustrating patient inclusion is shown in Figure 1. Baseline clinical features and the most relevant laboratory variables grouped according to admission BG levels are listed in Table 1. Overall, the mean age was 67.06 years (SD 16.4) and 57.1% of patients were male. The prevalence of diabetes was 18.9%.

**Table 1.** Baseline characteristics of SEMI-COVID-19 registry participants, according to admission blood glucose levels.

Variables	Patients with available data	Total	Admission BG <140 mg/dL	Admission BG 140–180 mg/dL	Admission BG >180 mg/dL	*p* Value
		*N* = 11,312	*N* = 8870	*N* = 1340	*N* = 1102	
			(78.41%)	(11.84%)	(9.74%)	
Demographics						
Mean age in years (SD)	11,312	67.06 (16.24)	65.2 (16.4)	73.34 (13.4)	74.31 (13.29)	<.001
Gender, male	11,312	6445 (57.1%)	5000 (56.4%)	752 (56.2%)	693 (62.9%)	<.001
Medical history, *n* (%)						
Diabetes	11,274	2125 (18.9%)	887 (10.0%)	512 (38.4%)	726 (66.0%)	<.001
Hypertension	11,291	5668 (50.2%)	3992 (45.1%)	879 (65.7%)	797 (72.5%)	<.001
Dyslipidaemia	11,292	4466 (39.6%)	3125 (35.3%)	713 (53.3%)	628 (57.1%)	<.001
Obesity	10,285	2191 (21.3%)	1606 (19.9%)	314 (25.7%)	271 (27.3%)	<.001
Dementia	11,281	1128 (10.0%)	747 (8.4%)	183 (13.7%)	198 (18.0%)	<.001
PAD	11,280	540 (4.8%)	353 (4.0%)	90 (6.7%)	97 (8.8%)	<.001
COPD	11,287	793 (7.0%)	568 (6.4%)	130 (9.7%)	95 (8.6%)	<.001
Atrial fibrillation	11,280	1257 (11.1%)	878 (9.9%)	193 (14.5%)	186 (16.9%)	<.001
CAD	11,206	490 (4.4%)	293 (3.3%)	105 (7.9%)	92 (8.4%)	<.001
Heart failure	11,285	830 (7.4%)	543 (6.1%)	145 (10.8%)	142 (12.9%)	<.001
CKD	11,279	689 (6.1%)	431 (4.9%)	121 (9.1%)	137 (12.5%)	<.001
Stroke	11,228	815 (7.2%)	552 (6.2%)	111 (8.3%)	152 (13.8%)	<.001
Dependent/frail patients	11,162	1829 (16.4%)	1214 (13.9%)	303 (23%)	312 (28.8%)	<.001
Previous diabetes treatment, *n* (%)						
Metformin	2124	1270 (59.8%)	531 (59.8%)	317 (62,2%)	422 (58.1%)	.360
iDPP4	2082	649 (31.2%)	207 (23.9%)	161 (32.2%)	281 (39.2%)	<.001
iSGLT2	2115	61 (2.9%)	28 (3.2%)	17 (3.3%)	16 (2.2%)	.424
arGLP1	2087	108 (5.2%)	45 (5.2%)	28 (5.6%)	35 (4.9%)	.872
Insulin	2112	121 (5.7%)	43 (4.9%)	38 (7.5%)	40 (5.6%)	.132
Charlson index	10,971	3 (1–5)	3 (1–5)	4 (3–6)	5 (4–7)	<.001
Blood count						
Lymphocyte count (×10^6^ L)	11,272	940 (700–1300)	990 (700–1300)	865 (600–1200)	810 (540–1200)	<.001
<800, *n* (%)		4417 (39.0%)	3244 (36.7%)	631 (47.2%)	542 (49.5%)	<.001
800–1200, *n* (%)		3611 (31.9%)	2929 (33.1%)	400 (29.9%)	282 (25.7%)	
>1200, *n* (%)		3244 (28.7%)	2667 (30.2%)	305 (22.8%)	272 (24.8%)	
Haemoglobin (g/dL)	11,297	13.7 (1.89)	13.80 (1.83)	13.49 (2.01)	13.34 (2.1)	<.001
Biochemistry						
Glucose (mg/dL)	11,312	127.04 (57.8)	106.08 (16.09)	156.84 (11.68)	259.57 (99.49)	<.001
Creatinine (mg/dL)	11,285	0.90(0.74–1.16)	0.81 (0.67–1.0)	0.88 (0.7.1.18)	0.94 (0.7–1.38)	<.001
Urea (mg/dL)	9082	37 (27–55)	35 (26–49)	44 (31–67)	55 (37.89)	<.001
Sodium (meq/L)	11,267	137.5 (4.68)	137.6 (4.2)	137.2 (5.2)	137.1 (6.8)	<.001
Potassium (meq/L)	11,046	4.12 (0,56)	4.1 (0.53)	4.1 (0.14)	4.3 (0.68)	<.001
LDH (U/L)	9817	313 (243–417)	308 (239–406)	336 (256–454)	333 (255–465)	<.001
<250, *n* (%)		2756 (24.4%)	2276 (29.3%)	270 (23.6%)	210 (23%)	<.001
250–400, *n* (%)		4331 (38.3%)	3471 (44.7%)	471 (41.2%)	389 (42.6%)	
>400, *n* (%)		2730 (24,1%)	2013 (25.9%)	403 (35.2%)	314 (34.4%)	
C-reactive protein (mg/L)	10,853	58.4 (18–126)	54 (17–116)	83 (24–160)	80 (26–161)	<.001
D-dimer (ng/mL)	8726	630 (359–1176)	588 (338–1063)	830 (454–1530)	945(511–2108)	<.001
<500, *n* (%)		3452 (30.5%)	2966 (43%)	290 (28.6%)	196 (24.1%)	<.001
500-1000, *n* (%)		2615 (23.1%)	2076 (31.1%)	306 (30.1%)	233 (28.7%)	
>1000, *n* (%)		2659 (23.5%)	1856 (26.9%)	419 (41.3%)	384 (47.2%)	
Serum ferritin (mcg/L)	4400	611 (291–1214)	598 (286–1200)	692 (317–1344)	639(305–1253)	.070
Interleukin-6 (pg/mL)	1621	29.8 (11.0–64.4)	29.8 (11.7–63.9)	33.6 (6.9–73.4)	25.4 (10.4–57.8)	.815
Treatment, *n* (%)						
Hydroxychloroquine	11,241	9680 (86.1%)	7729 (87.6%)	1089 (82%)	862 (78.8%)	<.001
Lopinavir/ritonavir	11,226	6986 (62.2%)	5633 (64.0%)	748 (56.3%)	605 (55.5%)	<.001
Tocilizumab	11,198	1000 (8.9%)	757 (8.6%)	130 (9.9%)	113 (10.4%)	.074
Systemic steroids	11,312	3950 (35.3%)	2875 (32.7%)	698 (45.9%)	467 (42.8%)	<.001

PAD: peripheral arterial disease; COPD: chronic obstructive pulmonary disease; CAD: coronary artery disease; CKD: chronic kidney disease; LDH: lactate dehydrogenase.

Quantitative variables are shown as mean (standard deviation) or median (25th percentile to 75th percentile).

Patients with higher admission BG levels were older, predominantly male, and more frequently had a prior history of diabetes, hypertension and other comorbidities. In addition, lymphocytes <800/mm^3^, LDH >400 U/L, D-dimer >1000 ng/mL, and elevated serum creatinine and C-reactive protein (CRP) levels were more frequent in patients with elevated admission BG. No differences were found in serum ferritin or interleukin-6 levels according to admission BG levels (Table 1).

Admission BG levels showed weak correlations with age (*ρ* = 0.252, *p*<.001), LDH (*ρ* = 0.156, *p*<.001), lymphocytes (*ρ*=–0.166, *p*<.001), D-dimer (*ρ* = 0.196, *p*<.001), serum creatinine (*ρ* = 0.213, *p*<.001) and CRP (*ρ* = 0.196, *p*<.001).

### Association between blood glucose and outcomes

**Figure 2.**
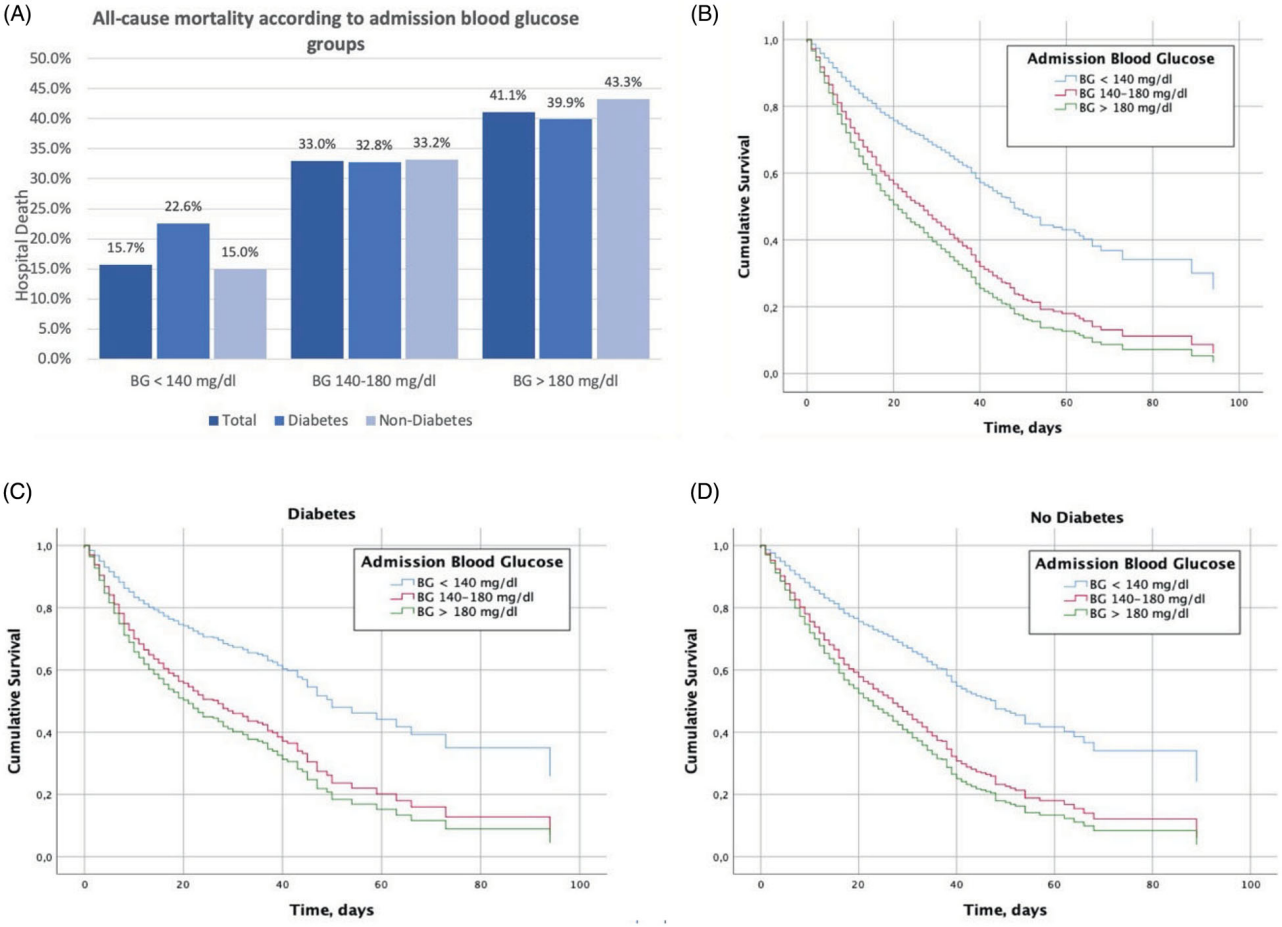
All-cause mortality (%) during hospitalization according to admission BG groups in all patients and based on the diabetes status, *p* value <.0001 (A). Kaplan–Meier’s curves according to admission BG levels in all patients (B) and in patients with diabetes (C) and without diabetes (D). BG <140 mg/dL (upper line), BG 140–180 mg/dL (middle line) and BG >180 mg/dL (lower line). Log rank *p*<.0001 for all curves.

In total, 2289 (20.2%) patients died during hospitalization. Main outcomes according to BG levels are showed in Table 2. All-cause mortality was higher in patients with admission BG levels >180 mg/dL (41.1%) compared to patients with levels of 140–180 mg/dL (33.0%) or <140 mg/dL (15.7%) (Figure 2(a)). Indeed, there was a gradual increase in all-cause mortality as admission BG levels increased, and there were no differences in mortality rates within each category of BG levels between patients with or without a previous history of diabetes (Figure 2(a)).

**Table 2.** Outcomes according to admission blood glucose levels.

Variables	Patients with available data	Total	Admission BG <140 mg/dL	Admission BG 140–180 mg/dL	Admission BG >180 mg/dL	*p* Value
		*N* = 11,312	*N* = 8870	*N* = 1340	*N* = 1102	
Outcomes, *n* (%)						
Death	11,312	2289 (20.2%)	1394 (15.7%)	442 (33.0%)	453 (41.1%)	<.001
Mechanical ventilation	11,217	1156 (10.2%)	790 (9%)	190 (14.3%)	176 (16.1%)	<.001
ICU admission	11,299	935 (8.3%)	668 (7.5%)	142 (10.6%)	125 (11.4%)	<.001
Composited endpoint	11,240	2978 (26.3%)	1911 (21.7%)	534 (40%)	533 (48.6%)	<.001
Length of stay, days	11,312	11.29 (9.39)	11.1 (9.1)	11.5 (9.8)	12.01 (10.6)	.011

ICU: intensive care unit; composited endpoint: death, mechanical ventilation and/or ICU admission.

Kaplan–Meier’s survival curves according to admission BG levels are shown in Figure 2(b) (log-rank *p*<.001). Similarly, survival curves after classifying the cohort according to diabetes status did not show any changes (log-rank *p*<.001) (Figure 2(c,d)).

After performing a multivariate stepwise Cox regression model adjusted for age, gender, hypertension, diabetes, COPD, dependency, lymphopaenia, anaemia (haemoglobin < 10 g/dL), serum creatinine, CRP > 60 mg/L, LDH > 400 U/L and D-dimer > 1000 ng/mL, elevated admission BG levels remained a significant predictor of death compared to BG <140 mg/dL, with the following findings: BG >180 mg/dL (HR 1.50, 95%CI: 1.31–1.73; *p*<.001) and BG 140–180 mg/dL (HR 1.48, 95%CI: 1.29–1.70; *p*<.001). In this model, age, male gender, hypertension, COPD, dependency/frailty, creatinine levels, CRP > 60 U/L and LDH > 400 U/L were also independently associated with all-cause mortality (Table 3).

**Table 3.** Association with all-cause in-hospital mortality.

	Univariate analysis		Multivariate analysis	
Variables	HR (95%CI)	*p* Value	HR (95%CI)	*p* Value
Age	1.067 (1.063–1.071)	<.001	1.055 (1.049–1.061)	<.001
Admission blood glucose				
<140 mg/dL	1		1	
140–180/dL	1.96 (1.75–2.19)	<.001	1.48 (1.29–1.70)	<.001
>180 mg/dL	2.30 (2.03–2.60)	<.001	1.50 (1.31–1.73)	<.001
Male	1.14 (1.05–1.24)	.002	1.15 (1.03–1.30)	.013
Hypertension	2.20 (2.01–2.41)	<.001	1.14 (1.01–1.29)	.029
Diabetes	1.63 (1.49–1.79)	<.001		.377
COPD	1.82 (1.61–2.05)	<.001	1.27 (1.08–1.49)	<.003
Dependency/frailty	3.21 (2.95–3.50)	<.001	1.58 (1.39–1.80)	<.001
Lymphopaenia <800 (×10^6^/L)	1.76 (1.58–1.96)	<.001		.868
Haemoglobin <10 g/dL	1.77 (1.52–2.07)	<.001		.630
Creatinine, mg/dL	1.25 (1.22–1.28)	<.001	1.33 (1.30–1.37)	<.001
CRP >60 mg/L	2.13 (1.94–2.33)	<.001	1.65 (1.47–1.85)	<.001
LDH >400 U/L	2.73 (2.38–3.14)	<.001	2.53 (2.51–2.97)	<.001
D-dimer >1000 ng/mL	2.60 (2.29–2.95)	<.001		.149

OR: odds ratio; COPD: chronic obstructive pulmonary disease; CRP: C-reactive protein; LDH: lactate dehydrogenase.

Adjusted multivariate Cox regression model. The model included all variables of medical history and laboratory findings.

LOS was slightly longer in patients with BG >180 mg/dL (12 days versus 11.5 days for BG 140–180 mg/dL and 11.1 days for BG <140 mg/dL; *p*<.011). Invasive or non-invasive ventilation and ICU admission were also associated with higher admission BG levels (Table 2). Finally, admission BG levels were independently related to the composite outcome (ICU admission, mechanical ventilation and/or death): BG >180 mg/dL (OR 2.02, 95%CI: 1.67–2.44; *p*<.001) and BG 140–180 mg/dL (OR 1.70, 95%CI: 1.43–2.02; *p*<.001) compared to BG <140 mg/dL (Table 4).

**Table 4.** Association with composite outcome (death, mechanical ventilation and/or ICU admission).

	Univariate analysis		Multivariate analysis	
Variables	OR (95%CI)	*p* Value	OR (95%CI)	*p* Value
Age	1.050 (1.046–1.053)	<.001	1.024 (1.019–1.029)	<.001
Admission blood glucose				
<140 mg/dL	1		1	
140–180 mg/dL	2.40 (2.13–2.71)	<.001	1.70 (1.43–2.02)	<.001
>180 mg/dL	3.41 (2.99–3.88)	<.001	2.02 (1.67–2.44)	<.001
Male	1.37 (1.25–1.49)	<.001	1.13 (0.99–1.28)	.066
Hypertension	2.08 (1.91–2.27)	<.001	NS	.226
Diabetes	1.86 (1.69–2.067)	<.001	NS	.618
COPD	2.34 (2.02–2.71)	<.001	1.59 (1.28–1.99)	<.001
Dependency/frailty	2.28 (2.96–3.64)	< .001	1.97 (1.66–2.32)	<.001
Lymphopaenia <800	2.69 (2.41–3.01)	<.001	1.85 (1.57–2.17)	<.001
Haemoglobin <10 g/dL	2.46 (2.03–2.99)	<.001	NS	.212
Creatinine, mg/dL	1.76 (1.66–1.88)	<.001	1.33 (1.24–1.42)	<.001
CRP >60 mg/L	2.61–2.38–2.85)	<.001	1.69 (1.63–2.35)	<.001
LDH >400 U/L	5.17 (4.51–5.92)	<.001	4.78 (3.97–5.75)	<.001
D-dime*r* > 1000 ng/mL	3.04 (2.69–3.43)	<.001	1.23 (1.05–1.43)	.008

OR: odds ratio; COPD: chronic obstructive pulmonary disease; CRP: C-reactive protein; LDH: lactate dehydrogenase.

Adjusted multivariate logistic regression model. The model included all variables of medical history and laboratory findings.

## Discussion

After analysing the data from 11,312 consecutive non-critically patients with confirmed COVID-19 admitted to Spanish hospitals, we found that the admission BG level was an independent predictor of all-cause mortality during hospitalization.

A small number of observational studies have analysed the relationship between glycaemic control and clinical outcomes in patients hospitalized due to COVID-19 with and without diabetes. All of them provide clinical evidence of a correlation between uncontrolled hyperglycaemia with poor prognosis and a particularly high mortality rate [3–7]. To date, our study has included by far the greatest number of valid patients. Furthermore, our study was conducted according to defined admission BG level groups, thus preventing the inclusion of patients with hyperglycaemia secondary to hospital management that may occur because of, for example, treatment with steroids. The above results were also confirmed in COVID-19 patients without a history of diabetes.

In a large retrospective study conducted by Zhu et al. [3] that included 7337 patients with COVID-19 hospitalized among 19 hospital in Hubei province, China, patients with diabetes had a significantly higher mortality rate (7.8% versus 2.7%; HR 1.49, 95%CI: 1.13–1.96; *p*=.005) than patients without diabetes. They also compared 528 patients with a history of poorly controlled diabetes (defined as BG >180 mg/dL) to 282 well-controlled patients (BG 70–180 mg/dL), finding that well-controlled patients had markedly lower all-cause mortality during hospitalization compared to poorly controlled patients (HR: 0.13, 95%CI: 0.04–0.44, *p*<.001). Similarly, our study also showed 1.63-fold increase in mortality in patients with diabetes on the univariate analysis. However, Zhu et al. did not include patients without diabetes in their study, so the effect of hyperglycaemia in the non-diabetic population with COVID-19 cannot be analysed.

A retrospective observational study (*n* = 1122) by Bode et al. [5] from 88 hospitals in the USA studied the clinical outcomes of 451 patients with diabetes (HbA1c ≥ 6.5%) and/or uncontrolled hyperglycaemia (defined as ≥2 BG values > 180 mg/dL in a 24-hour period with HbA1c ≤6.5% or no A1c available) using data abstracted from Glytec’s data warehouse. Among the 570 patients who died or were discharged, the mortality rate was 28.8% in the group with diabetes and/or uncontrolled hyperglycaemia (*n* = 184) compared to 6.2% in the control group (*n* = 386) (*p*<.001). The mortality rate was particularly high among patients with uncontrolled hyperglycaemia without diabetes (41.7%) compared to patients with diabetes (14.8%) (*p*<.001). This finding is consistent with our results (a mortality rate of 43.3% in non-diabetic patients with admission BG >180 mg/dL). Overall, these findings suggest that stress hyperglycaemia could play a crucial role in the prognosis of patients hospitalized with COVID-19.

Two observational studies conducted in Wuhan, China compared the relationship between hyperglycaemia and outcomes in patients hospitalized with confirmed COVID-19. Zhang et al. [6] evaluated the relationship between hyperglycaemia and outcomes in patients (*n* = 166) hospitalized with confirmed COVID-19 with diabetes and secondary hyperglycaemia classified into three groups: control (group 1: no history of diabetes and basal BG <126 mg/dL), secondary hyperglycaemia (group 2: no history of diabetes and basal BG ≥126 mg/dL and HbA1c <6.5%) and diabetes (group 3: basal BG ≥126 mg/dL and history of diabetes or HbA1c ≥6.5%). The mortality rates in groups 2 and 3 were significantly higher compared to group 1 (21.3%, 14.3% and 9.5%, respectively; *p*<.05 for both). The composite outcome of ICU admission, use of either invasive or non-invasive mechanical ventilation, or death occurred in 38.1% of patients in group 2 and in 27.9% of patients of group 3. After adjusted for confounding variables, the OR for composite outcomes was 5.47 (95%CI 1.56–19.82) and 2.61 (95%CI 0.86–7.88) in groups 2 and 3 compared to group 1. These results suggest that stress hyperglycaemia is also related to poor prognosis and mortality in patients infected by SARS-CoV-2.

Wu et al. [7] collected data on 2041 patients admitted to two medical centres. They compared non-critical patients at admission (*n* = 1690) to critical patients at the time of admission or at the time of transition from the non-critical group (*n* = 697). Elevated admission BG levels (defined as admission BG ≥110 mg/dL) were an independent risk factor for progression to critical status (ICU admission, mechanical ventilation, compromised hemodynamic) or death among non-critical patients (OR 1.30; 95%CI 1.03–1.63, *p*=.026). Higher median BG levels after a non-critical patient becomes critical were also independently associated with higher in-hospital mortality (OR 2.39; 95%CI 1.41–4.07, *p*=.001). Similar to our results, hyperglycaemia but not diabetes was associated to worse outcomes and death on the multivariate analysis.

Sardu et al. [4] studied 59 patients with COVID-19 hospitalized in two Italian hospitals. Patients were divided into a hyperglycaemic group (*n* = 25), defined as patients with admission BG >140 mg/dL, and a normoglycaemic group (*n* = 34). In the hyperglycaemic group, 18 (72%) patients had prior history of diabetes and 15 (60%) were treated with an insulin infusion until they reached a BG level <140 mg/dL. At baseline, interlukin-6 and D-dimer levels were also significantly higher in the hyperglycaemic group than in the normoglycaemic group (*p*<.001). After adjusting the model for confounding variables, patients with hyperglycaemia treated with an insulin infusion had a lower risk of complications than patients who did not receive an insulin infusion. Additionally, interleukin-6 and D-dimer levels were reduced after treating the hyperglycaemia. The authors concluded that optimal glycaemic control during hospitalization could be associated with reduced risk of severe disease and death.

Another recent study showed that COVID-19 patients with recently diagnosed diabetes had the highest risk of all-cause mortality compared to those who had diabetes for a longer time (HbA1c ≥6.5%), hyperglycaemia or normoglycaemia [18]. Wang et al. [10] showed, in a relatively small sample (*n* = 695), that fasting BG >126 mg/dL at admission was an independent predictor for 28-day mortality in patients without a previous diagnosis of diabetes.

Our study, which analyses the largest number of patients of any study to date, is consistent with all these results and reinforces the strong association found between hyperglycaemia and in-hospital mortality in non-critically patients hospitalized with COVID-19, independently of prior history of diabetes. Acute hyperglycaemia occurs in about 22% of patients hospitalized for COVID-19, while 18.9% of patients had diabetes, but 10% of them had admission BG <140 mg/dL. Other data registry shows that acute hyperglycaemia occurs in about 50%, while the prevalence of diabetes is about 7% [19].

One question that remains to be answered is whether hyperglycaemia plays any role in the physiopathology of the disease or if it is just an inflammatory bystander. Apart from the glycosylation of ACE2 receptors that facilitates virus binding and the inflammatory process that increases insulin resistance, the hypoxia that is normally present in patients with COVID-19 is frequently accompanied by disordered cellular glucose metabolism. Under anaerobic conditions, glucose ferments into lactate, which produces a limited amount of adenosine triphosphate (ATP). Hypoxia and ATP depletion cause an elevation of blood lactate and LDH levels. In our study, elevation of the LDH level was also associated with mortality according to BG levels, a finding that is consistent with the mechanism described above. This finding suggests that an early imbalance in glucose metabolism could be involved in a crucial manner in the physiopathology of the viral respiratory infection. Only one very small study suggests that supplemental oxygen at the earliest stages of COVID-19 could be useful in correcting an anaerobic glucose metabolism imbalance [20]. Adequate oxygen delivery and BG monitoring should be carried out for patients who are asked to remain at home in the early stages of the infection in order to prevent clinical deterioration. Early correction of hyperglycaemia in the course of COVID-19 could result in a decrease in the release of inflammatory cytokines and a reduction in the virus’ ACE binding capacity, consequently resulting in better outcomes [11].

Both strategies – screening for hyperglycaemia in patients without diabetes and early treatment of hyperglycaemia – should be mandatory in the management of patients hospitalized with COVID-19 [21]. Unfortunately, insufficient evidence is available on the benefits of strict glycaemic control in patients hospitalized with COVID-19 due to the fact that glycaemic management was underestimated during outbreak and the difficulties of multiple daily insulin injections and frequent point-of-care glucose testing in areas with high burden of COVID-19 patients [22,23]. Thus, to date, early glycaemic control may be a suitable therapeutic option to reduce the poor outcomes in hospitalized hyperglycaemic COVID-19 patients with or without a previous diabetes diagnosis [24].

This study has several limitations. First, it is an observational retrospective cohort study conducted during an outbreak, so there may be residual or unmeasured confounding factors. Second, most patients did not have a HbA1c measurement and as such, some patients classified as non-diabetic could have unknown diabetes. Third, the registry is missing data on some relevant inflammatory variables such as interleukin-6, D-dimer and serum ferritin. Finally, time from hospital admission to ICU admission was not available.

On the other hand, as a strength, our registry is the largest available cohorts of non-critically ill hospitalized patients with confirmed COVID-19 in contrast to other studies focuses on critically patients and it includes data from over 11,000 patients on admission BG levels before starting any treatment.

In conclusion, our study found that admission hyperglycaemia was an independent predictor of progression to critical condition and all-cause mortality in non-critically patients hospitalized with COVID-19. Moreover, this finding is independent of a prior history of diabetes. These results provided a simple and practical way to stratify risk of death in hospitalized patients with COVID-19. Hence, admission hyperglycaemia should not be overlooked, but rather detected and appropriately treated to improve the outcomes of COVID-19 patients with and without diabetes.

## Data Availability

The steering committee of the Spanish SEMI-COVID-19 Registry will consider reasonable requests for the sharing of data. Requests should be made to the corresponding author.

## Appendix

### List of the SEMI-COVID-19 network members

*Coordinator of the SEMI-COVID-19 Registry:* José Manuel Casas Rojo.

*SEMI-COVID-19 Scientific Committee Members:* José Manuel Casas Rojo, José Manuel Ramos Rincón, Carlos Lumbreras Bermejo, Jesús Millán Núñez-Cortés, Juan Miguel Antón Santos, Ricardo Gómez Huelgas.

*SEMI-COVID-19 Registry Coordinating Center:* S & H Medical Science Service.

### Members of the SEMI-COVID-19 Group

**H. U. 12 de Octubre. Madrid**

Paloma Agudo de Blas, Coral Arévalo Cañas, Blanca Ayuso, José Bascuñana Morejón, Samara Campos Escudero, María Carnevali Frías, Santiago Cossio Tejido, Borja de Miguel Campo, Carmen Díaz Pedroche, Raquel Diaz Simon, Ana García Reyne, Lucia Jorge Huerta, Antonio Lalueza Blanco, Jaime Laureiro Gonzalo, Carlos Lumbreras Bermejo, Guillermo Maestro de la Calle, Barbara Otero Perpiña, Diana Paredes Ruiz, Marcos Sánchez Fernández, Javier Tejada Montes.

**H. U. Gregorio Marañón. Madrid**

Laura Abarca Casas, Álvaro Alejandre de Oña, Rubén Alonso Beato, Leyre Alonso Gonzalo, Jaime Alonso Muñoz, Crhistian Mario Amodeo Oblitas, Cristina Ausín García, Marta Bacete Cebrián, Jesús Baltasar Corral, Maria Barrientos Guerrero, Alejandro Bendala Estrada, María Calderón Moreno, Paula Carrascosa Fernández, Raquel Carrillo, Sabela Castañeda Pérez, Eva Cervilla Muñoz, Agustín Diego Chacón Moreno, Maria Carmen Cuenca Carvajal, Sergio de Santos, Andrés Enríquez Gómez, Eduardo Fernández Carracedo, María Mercedes Ferreiro-Mazón Jenaro, Francisco Galeano Valle, Alejandra Garcia, Irene Garcia Fernandez-Bravo, María Eugenia García Leoni, Maria Gomez Antunez, Candela González San Narciso, Anthony Alexander Gurjian, Lorena Jiménez Ibáñez, Cristina Lavilla Olleros, Cristina Llamazares Mendo, Sara Luis García, Víctor Mato Jimeno, Clara Millán Nohales, Jesús Millán Núñez-Cortés, Sergio Moragón Ledesma, Antonio Muiño Miguez, Cecilia Muñoz Delgado, Lucía Ordieres Ortega, Susana Pardo Sánchez, Alejandro Parra Virto, María Teresa Pérez Sanz, Blanca Pinilla Llorente, Sandra Piqueras Ruiz, Guillermo Soria Fernández-Llamazares, María Toledano Macías, Neera Toledo Samaniego, Ana Torres do Rego, Maria Victoria Villalba Garcia, Gracia Villarreal, María Zurita Etayo.

**Hospital Universitari de Bellvitge. L'Hospitalet de Llobregat**

Xavier Corbella, Abelardo Montero, Jose María Mora-Luján.

**C. H. U. de Albacete. Albacete**

Jose Luis Beato Pérez, Maria Lourdes Sáez Méndez.

**H. U. La Paz-Cantoblanco-Carlos III. Madrid**

Jorge Álvarez Troncoso, Francisco Arnalich Fernández, Francisco Blanco Quintana, Carmen Busca Arenzana, Sergio Carrasco Molina, Aranzazu Castellano Candalija, Germán Daroca Bengoa, Alejandro de Gea Grela, Alicia de Lorenzo Hernández, Alejandro Díez Vidal, Carmen Fernández Capitán, Maria Francisca García Iglesias, Borja González Muñoz, Carmen Rosario Herrero Gil, Juan María Herrero Martínez, Víctor Hontañón, Maria Jesús Jaras Hernández, Carlos Lahoz, Cristina Marcelo Calvo, Juan Carlos Martín Gutiérrez, Monica Martinez Prieto, Elena Martínez Robles, Araceli Menéndez Saldaña, Alberto Moreno Fernández, Jose Maria Mostaza Prieto, Ana Noblejas Mozo, Carlos Manuel Oñoro López, Esmeralda Palmier Peláez, Marina Palomar Pampyn, Maria Angustias Quesada Simón, Juan Carlos Ramos Ramos, Luis Ramos Ruperto, Aquilino Sánchez Purificación, Teresa Sancho Bueso, Raquel Sorriguieta Torre, Clara Itziar Soto Abanedes, Yeray Untoria Tabares, Marta Varas Mayoral, Julia Vásquez Manau.

**Complejo Asistencial de Segovia. Segovia**

Eva María Ferreira Pasos, Daniel Monge Monge, Alba Varela García.

**H. U. Puerta de Hierro. Majadahonda**

María Álvarez Bello, Ane Andrés Eisenhofer, Ana Arias Milla, Isolina Baños Pérez, Javier Bilbao Garay, Silvia Blanco Alonso, Jorge Calderón Parra, Alejandro Callejas Díaz, José María Camino Salvador, Mª Cruz Carreño Hernández, Valentín Cuervas-Mons Martínez, Sara de la Fuente Moral, Miguel del Pino Jimenez, Alberto Díaz de Santiago, Itziar Diego Yagüe, Ignacio Donate Velasco, Ana María Duca, Pedro Durán del Campo, Gabriela Escudero López, Esther Expósito Palomo, Ana Fernández Cruz, Esther Fiz Benito, Andrea Fraile López, Amy Galán Gómez, Sonia García Prieto, Claudia García Rodríguez-Maimón, Miguel Ángel García Viejo, Javier Gómez Irusta, Edith Vanessa Gutiérrez Abreu, Isabel Gutiérrez Martín, Ángela Gutiérrez Rojas, Andrea Gutiérrez Villanueva, Jesús Herráiz Jiménez, Pedro Laguna del Estal, Mª Carmen Máinez Sáiz, Cristina Martín Martín, María Martínez Urbistondo, Fernando Martínez Vera, Susana Mellor Pita, Patricia Mills Sánchez, Esther Montero Hernández, Alberto Mora Vargas, Cristina Moreno López, Alfonso Ángel-Moreno Maroto, Victor Moreno-Torres Concha, Ignacio Morrás De La Torre, Elena Múñez Rubio, Ana Muñoz Gómez, Rosa Muñoz de Benito, Alejandro Muñoz Serrano, Jose María Palau Fayós, Ilduara Pintos Pascual, Antonio Ramos Martínez, Isabel Redondo Cánovas del Castillo, Alberto Roldán Montaud, Lucía Romero Imaz, Yolanda Romero Pizarro, Mónica Sánchez Santiuste, David Sánchez Órtiz, Enrique Sánchez Chica, Patricia Serrano de la Fuente, Pablo Tutor de Ureta, Ángela Valencia Alijo, Mercedes Valentín-Pastrana Aguilar, Juan Antonio Vargas Núñez, Jose Manuel Vázquez Comendador, Gema Vázquez Contreras, Carmen Vizoso Gálvez.

**H. Miguel Servet. Zaragoza**

Gonzalo Acebes Repiso, Uxua Asín Samper, María Aranzazu Caudevilla Martínez, José Miguel García Bruñén, Rosa García Fenoll, Jesús Javier González Igual, Laura Letona Giménez, Mónica Llorente Barrio, Luis Sáez Comet.

**H. U. La Princesa. Madrid**

María Aguilera García, Ester Alonso Monge, Jesús Álvarez Rodríguez, Claudia Alvarez Varela, Miquel Berniz Gòdia, Marta Briega Molina, Marta Bustamante Vega, Jose Curbelo, Alicia de las Heras Moreno, Ignacio Descalzo Godoy, Alexia Constanza Espiño Alvarez, Ignacio Fernández Martín-Caro, Alejandra Franquet López-Mosteiro, Gonzalo Galvez Marquez, María J. García Blanco, Yaiza García del Álamo Hernández, Clara García-Rayo Encina, Noemí Gilabert González, Carolina Guillamo Rodríguez, Nicolás Labrador San Martín, Manuel Molina Báez, Carmen Muñoz Delgado, Pedro Parra Caballero, Javier Pérez Serrano, Laura Rabes Rodríguez, Pablo Rodríguez Cortés, Carlos Rodriguez Franco, Emilia Roy-Vallejo, Monica Rueda Vega, Aresio Sancha Lloret, Beatriz Sánchez Moreno, Marta Sanz Alba, Jorge Serrano Ballester, Alba Somovilla, Carmen Suarez Fernández, Macarena Vargas Tirado, Almudena Villa Marti.

**H. U. de A Coruña. A Coruña**

Alicia Alonso Álvarez, Olaya Alonso Juarros, Ariadna Arévalo López, Carmen Casariego Castiñeira, Ana Cerezales Calviño, Marta Contreras Sánchez, Ramón Fernández Varela, Santiago J. Freire Castro, Ana Padín Trigo, Rafael Prieto Jarel, Fátima Raad Varea, Laura Ramos Alonso, Francisco Javier Sanmartín Pensado, David Vieito Porto.

**H. Clinico San Carlos. Madrid**

Inés Armenteros Yeguas, Javier Azaña Gómez, Julia Barrado Cuchillo, Irene Burruezo López, Noemí Cabello Clotet, Alberto E. Calvo Elías, Elpidio Calvo Manuel, Carmen María Cano de Luque, Cynthia Chocron Benbunan, Laura Dans Vilan, Ester Emilia Dubon Peralta, Vicente Estrada Pérez, Santiago Fernandez-Castelao, Marcos Oliver Fragiel Saavedra, José Luis García Klepzig, Maria del Rosario Iguarán Bermúdez, Esther Jaén Ferrer, Rubén Ángel Martín Sánchez, Manuel Méndez Bailón, Maria José Nuñez Orantos, Carolina Olmos Mata, Eva Orviz García, David Oteo Mata, Cristina Outon González, Juncal Perez-Somarriba, Pablo Pérez Mateos, Maria Esther Ramos Muñoz, Xabier Rivas Regaira, Iñigo Sagastagoitia Fornie, Alejandro Salinas Botrán, Miguel Suárez Robles, Maddalena Elena Urbano, Miguel Villar Martínez.

**H. Infanta Sofía. S. S. de los Reyes**

Rafael del Castillo Cantero, Rebeca Fuerte Martínez, Arturo Muñoz Blanco, José Francisco Pascual Pareja, Isabel Perales Fraile, Isabel Rábago Lorite, Llanos Soler Rangel, Inés Suárez García, Jose Luis Valle López.

**Hospital Universitario Dr. Peset. Valencia**

Juan Alberto Aguilera Ayllón, Arturo Artero Mora, María del Mar Carmona Martín, María José Fabiá Valls, Maria de Mar Fernández Garcés, Ana Belén Gómez Belda, Ian López Cruz, Manuel Madrazo López, Elisabet Mateo Sanchis, Jaume Micó Gandia, Laura Piles Roger, Adela Maria Pina Belmonte, Alba Viana García.

**Hospital Clínico de Santiago. Santiago de Compostela**

Maria del Carmen Beceiro Abad, Maria Aurora Freire Romero, Sonia Molinos Castro, Emilio Manuel Paez Guillan, María Pazo Nuñez, Paula Maria Pesqueira Fontan.

**H. U. Ramón y Cajal. Madrid**

Luis Fernando Abrego Vaca, Ana Andréu Arnanz, Octavio Arce García, Marta Bajo González, Pablo Borque Sanz, Alberto Cozar Llisto, Sonia de Pedro Baena, Beatriz Del Hoyo Cuenda, María Alejandra Gamboa Osorio, Isabel García Sánchez, Andrés González García, Oscar Alberto López Cisneros, Miguel Martínez Lacalzada, Borja Merino Ortiz, Jimena Rey-García, Elisa Riera González, Cristina Sánchez Díaz, Grisell Starita Fajardo, Cecilia Suárez Carantoña, Adrian Viteri Noel, Svetlana Zhilina Zhilina.

**Hospital Royo Villanova. Zaragoza**

Nicolás Alcalá Rivera, Anxela Crestelo Vieitez, Esther del Corral, Jesús Díez Manglano, Isabel Fiteni Mera, Maria del Mar Garcia Andreu, Martin Gerico Aseguinolaza, Claudia Josa Laorden, Raul Martinez Murgui, Marta Teresa Matía Sanz.

**H. U. Infanta Cristina. Parla**

Juan Miguel Antón Santos, Ana Belén Barbero Barrera, Coralia Bueno Muiño, Ruth Calderón Hernaiz, Irene Casado Lopez, José Manuel Casas Rojo, Andrés Cortés Troncoso, Mayte de Guzmán García-Monge, Francesco Deodati, Gonzalo García Casasola Sánchez, Elena Garcia Guijarro, Davide Luordo, María Mateos González, Jose A Melero Bermejo, Lorea Roteta García, Elena Sierra Gonzalo, Javier Villanueva Martínez.

**H. de Cabueñes. Gijón**

Ana María Álvarez Suárez, Carlos Delgado Vergés, Rosa Fernandez-Madera Martínez, Eva Fonseca Aizpuru, Alejandro Gómez Carrasco, Cristina Helguera Amezua, Juan Francisco López Caleya, María del Mar Martínez López, Aleida Martínez Zapico, Carmen Olabuenaga Iscar, María Luisa Taboada Martínez, Lara María Tamargo Chamorro.

**Hospital de Urduliz Alfredo Espinosa. Urdúliz**

María Aparicio López, Asier Aranguren Arostegui, Paula Arriola Martínez, Gorka Arroita Gonzalez, Mª Soledad Azcona Losada, Miriam García Gómez, Eduardo Garcia Lopez, Amalur Iza Jiménez, Alazne Lartategi Iraurgi, Esther Martinez Becerro, Itziar Oriñuela González, Isabel María Portales Fernández, Pablo Ramirez Sánchez, Beatriz Ruiz Estévez, Cristian Vidal Núñez.

**Hospital Regional Universitario de Málaga. Málaga**

Mª Mar Ayala Gutiérrez, Rosa Bernal López, José Bueno Fonseca, Verónica Andrea Buonaiuto, Luis Francisco Caballero Martínez, Lidia Cobos Palacios, Clara Costo Muriel, Francis de Windt, Ana Teresa Fernandez-Truchaud Christophel, Paula García Ocaña, Ricardo Gómez Huelgas, Javier Gorospe García, Maria Dolores López Carmona, Pablo López Quirantes, Almudena López Sampalo, Elizabeth Lorenzo Hernández, Juan José Mancebo Sevilla, Jesica Martin Carmona, Luis Miguel Pérez-Belmonte, Araceli Pineda Cantero, Michele Ricci, Jaime Sanz Cánovas

**H. Santa Marina. Bilbao**

Maria Areses Manrique, Ainara Coduras Erdozain, Ane Elbire Labirua-Iturburu Ruiz.

**H. Nuestra Señora del Prado. Talavera de la Reina**

Sonia Casallo Blanco, Jeffrey Oskar Magallanes Gamboa.

**Hospital HLA Moncloa. Madrid**

Guillermo Estrada, Teresa Garcia Delange, Isabel Jimenez Martinez, Carmen Martinez Cilleros, Nuria Parra Arribas.

**H. del Henares. Coslada**

Jesús Ballano Rodríguez-Solís, Luis Cabeza Osorio, María del Pilar Fidalgo Montero, Mª Isabel Fuentes Soriano, Erika Esperanza Lozano Rincon, Ana Martín Hermida, Jesus Martinez Carrilero, Jose Angel Pestaña Santiago, Manuel Sánchez Robledo, Patricia Sanz Rojas, Nahum Jacobo Torres Yebes, Vanessa Vento.

**H. U. Torrevieja. Torrevieja**

Julio César Blázquez Encinar.

**H. U. La Fe. Valencia**

Dafne Cabañero, María Calabuig Ballester, Pascual Císcar Fernández, Ricardo Gil Sánchez, Marta Jiménez Escrig, Cristina Marín Amela, Laura Parra Gómez, Carlos Puig Navarro, José Antonio Todolí Parra.

**H. San Pedro. Logroño**

Diana Alegre González, Irene Ariño Pérez de Zabalza, Sergio Arnedo Hernández, Jorge Collado Sáenz, Beatriz Dendariena, Marta Gómez del Mazo, Iratxe Martínez de Narvajas Urra, Sara Martínez Hernández, Estela Menendez Fernández, Jose Luís Peña Somovilla, Elisa Rabadán Pejenaute.

**Hospital Universitario Ntra Sra Candelaria. Santa Cruz de Tenerife**

Lucy Abella, Andrea Afonso Díaz, Selena Gala Aguilera Garcia, Marta Bethencourt Feria, Eduardo Mauricio Calderón Ledezma, Sara Castaño Perez, Guillermo Castro Gainett, José Manuel del Arco Delgado, Joaquín Delgado Casamayor, Diego Garcia Silvera, Alba Gómez Hidalgo, Marcelino Hayek Peraza, Carolina Hernández Carballo, Rubén Hernández Luis, Francisco Javier Herrera Herrera, Maria del Mar Lopez Gamez, Julia Marfil Daza, María José Monedero Prieto, María Blanca Monereo Muñoz, María de la Luz Padilla Salazar, Daniel Rodríguez Díaz, Alicia Tejera, Laura Torres Hernández.

**H. U. San Juan de Alicante. San Juan de Alicante**

David Balaz, David Bonet Tur, Carles García Cervera, David Francisco García Núñez, Vicente Giner Galvañ, Angie Gómez Uranga, Javier Guzmán Martínez, Isidro Hernández Isasi, Lourdes Lajara Villar, Juan Manuel Núñez Cruz, Sergio Palacios Fernández, Juan Jirge Peris García, Andrea Riaño Pérez, José Miguel Seguí Ripoll, Philip Wikman-Jorgensen.

**H. U. San Agustin. Avilés**

Andrea Álvarez García, Víctor Arenas García, Alba Barragán Mateos, Demelsa Blanco Suárez, María Caño Rubia, Jaime Casal Álvarez, David Castrodá Copa, José Ferreiro Celeiro, Natalia García Arenas, Raquel García Noriega, Joaquin Llorente García, Irene Maderuelo Riesco, Paula Martinez Garcia, Maria Jose Menendez Calderon, Diego Eduardo Olivo Aguilar, Marta Nataya Solís Marquínez, Luis Trapiella Martínez, Andrés Astur Treceño García, Juan Valdés Bécares.

**H. de Mataró. Mataró**

Raquel Aranega González, Ramon Boixeda, Carlos Lopera Mármol, Marta Parra Navarro, Ainhoa Rex Guzmán, Aleix Serrallonga Fustier.

**H. U. Son Llàtzer. Palma de Mallorca**

Andrés de la Peña Fernández, Almudena Hernández Milián.

**H. Juan Ramón Jiménez. Huelva**

Francisco Javier Bejarano Luque, Francisco Javier Carrasco-Sánchez, Mercedes de Sousa Baena, Jaime Díaz Leal, Aurora Espinar Rubio, Maria Franco Huertas, Juan Antonio García Bravo, Andrés Gonzalez Macías, Encarnación Gutiérrez Jiménez, Alicia Hidalgo Jiménez, Constantino Lozano Quintero, Carmen Mancilla Reguera, Francisco Javier Martínez Marcos, Francisco Muñoz Beamud, María Pérez Aguilera, Virginia Rodríguez Castaño, Álvaro Sánchez de Alcázar del Río, Leire Toscano Ruiz.

**H. U. Reina Sofía. Córdoba**

Antonio Pablo Arenas de Larriva, Pilar Calero Espinal, Javier Delgado Lista, María Jesús Gómez Vázquez, Jose Jiménez Torres, Laura Martín Piedra, Javier Pascual Vinagre, María Elena Revelles Vílchez, Juan Luis Romero Cabrera, José David Torres Peña.

**H. Moisès Broggi. Sant Joan Despí**

Jose Loureiro Amigo, Melani Pestaña Fernández, Nicolas Rhyman, Nuria Vázquez Piqueras.

**H. U. Virgen de las Nieves. Granada**

Pablo Conde Baena, Joaquin Escobar Sevilla, Laura Gallo Padilla, Patricia Gómez Ronquillo, Pablo González Bustos, María Navío Botías, Jessica Ramírez Taboada, Mar Rivero Rodrígez.

**H. San Juan de la Cruz. Úbeda**

Marcos Guzmán Garcia, Francisco Javier Vicente Hernández.

**Hospital Costa del Sol. Marbella**

Victoria Augustín Bandera, María Dolores Martín Escalante.

**Hospital Infanta Margarita. Cabra**

María Esther Guisado Espartero, Lorena Montero Rivas, Maria de la Sierra Navas Alcántara, Raimundo Tirado-Miranda.

**Complejo Asistencial Universitario de León. León**

Rosario Maria García Die, Manuel Martin Regidor, Angel Luis Martínez Gonzalez, Alberto Muela Molinero, Raquel Rodríguez Díez, Beatriz Vicente Montes.

**Hospital Clinic Barcelona. Barcelona**

Júlia Calvo Jiménez, Aina Capdevila Reniu, Irene Carbonell De Boulle, Emmanuel Coloma Bazán, Joaquim Fernández Solà, Cristina Gabara Xancó, Joan Ribot Grabalosa, Olga Rodríguez Núñez.

**Hospital Marina Baixa. Villajoyosa**

Javier Ena, Jose Enrique Gómez Segado.

**C. H. U. de Ferrol. Ferrol**

Hortensia Alvarez Diaz, Tamara Dalama Lopez, Estefania Martul Pego, Carmen Mella Pérez, Ana Pazos Ferro, Sabela Sánchez Trigo, Dolores Suarez Sambade, Maria Trigas Ferrin, Maria del Carmen Vázquez Friol, Laura Vilariño Maneiro.

**Hospital Insular de Gran Canaria. Las Palmas G. C**

Carlos Jorge Ripper.

**Hospital del Tajo. Aranjuez**

Ruth Gonzalez Ferrer, Raquel Monsalvo Arroyo.

**H. U. Marqués de Valdecilla. Santander**

Marta Fernández-Ayala Novo, José Javier Napal Lecumberri, Nuria Puente Ruiz, Jose Riancho, Isabel Sampedro Garcia.

**Hospital Torrecárdenas. Almería**

Luis Felipe Díez García, Iris El Attar Acedo, Bárbara Hernandez Sierra, Carmen Mar Sánchez Cano.

**H. U. Severo Ochoa. Leganés**

Yolanda Casillas Viera, Lucía Cayuela Rodríguez, Carmen de Juan Alvarez, Gema Flox Benitez, Laura García Escudero, Juan Martin Torres, Patricia Moreira Escriche, Susana Plaza Canteli, M Carmen Romero Pérez.

**Hospital Valle del Nalón. Riaño (Langreo)**

Sara Fuente Cosío, César Manuel Gallo Álvaro, Julia Lobo García, Antía Pérez Piñeiro.

**H. U. del Vinalopó. Elche**

Francisco Amorós Martínez, Erika Ascuña Vásquez, Jose Carlos Escribano Stablé, Adriana Hernández Belmonte, Ana Maestre Peiró, Raquel Martínez Goñi, M. Carmen Pacheco Castellanos, Bernardino Soldan Belda, David Vicente Navarro.

**Hospital Alto Guadalquivir. Andújar**

Begoña Cortés Rodríguez.

**H. Francesc de Borja. Gandia**

Alba Camarena Molina, Simona Cioaia, Anna Ferrer Santolalia, José María Frutos Pérez, Eva Gil Tomás, Leyre Jorquer Vidal, Marina Llopis Sanchis, Mari Ángeles Martínez Pascual, Alvaro Navarro Batet, Mari Amparo Perea Ribis, Ricardo Peris Sanchez, José Manuel Querol Ribelles, Silvia Rodriguez Mercadal, Ana Ventura Esteve.

**H. G. U. de Castellón. Castellón de la Plana**

Jorge Andrés Soler, Marián Bennasar Remolar, Alejandro Cardenal Álvarez, Daniela Díaz Carlotti, María José Esteve Gimeno, Sergio Fabra Juana, Paula García López, María Teresa Guinot Soler, Daniela Palomo de la Sota, Guillem Pascual Castellanos, Ignacio Pérez Catalán, Celia Roig Martí, Paula Rubert Monzó, Javier Ruiz Padilla, Nuria Tornador Gaya, Jorge Usó Blasco.

**H. Santa Bárbara. Soria**

Marta Leon Tellez.

**C. A. U. de Salamanca. Salamanca**

Gloria María Alonso Claudio, Víctor Barreales Rodríguez, Cristina Carbonell Muñoz, Adela Carpio Pérez, María Victoria Coral Orbes, Daniel Encinas Sánchez, Sandra Inés Revuelta, Miguel Marcos Martín, José Ignacio Martín González, José Ángel Martín Oterino, Leticia Moralejo Alonso, Sonia Peña Balbuena, María Luisa Pérez García, Ana Ramon Prados, Beatriz Rodríguez-Alonso, Ángela Romero Alegría, Maria Sanchez Ledesma, Rosa Juana Tejera Pérez.

**H. Virgen de la Salud. Toledo**

Ana Maria Alguacil Muñoz, Marta Blanco Fernández, Veronica Cano, Ricardo Crespo Moreno, Fernando Cuadra Garcia-Tenorio, Blanca Díaz-Tendero Nájera, Raquel Estévez González, María Paz García Butenegro, Alberto Gato Díez, Verónica Gómez Caverzaschi, Piedad María Gómez Pedraza, Julio González Moraleja, Raúl Hidalgo Carvajal, Patricia Jiménez Arandq, Raquel Labra González, Áxel Legua Caparachini, Pilar Lopez Castañeyra, Agustín Lozano Ancin, Jose Domingo Martin Garcia, Cristina Morata Romero, María Jesús Moya Saiz, Helena Moza Moríñigo, Gemma Muñiz Nicolás, Enriqueta Muñoz Platon, Elena Ortiz Ortiz, Raúl Perea Rafael, Pilar Redondo Galán, María Antonia Sepulveda Berrocal, Pilar Toledano Sierra, Jesús Vázquez Clemente, Carmen Yera Bergua.

**H. U. de Canarias. Santa Cruz de Tenerife**

Julio Cesar Alvisa Negrin, José Fernando Armas González, Lourdes González Navarrete, Iballa Jiménez, María Candelaria Martín González, Miguel Nicolas Navarrete Lorite, Paula Ortega Toledo, Onán Pérez Hernández, Alina Pérez Ramírez.

**H. de Poniente. Almería**

Juan Antonio Montes Romero, Encarna Sánchez Martín, Jose Luis Serrano Carrillo de Albornoz, Manuel Jesus Soriano Pérez.

**H. U. Lucus Augusti. Lugo**

Raquel Gómez Méndez, Ana Rodríguez Álvarez.

**H. San Pedro de Alcántara. Cáceres**

Angela Agea Garcia, Javier Galán González, Luis Gámez Salazar, Eva Garcia Sardon, Antonio González Nieto, Itziar Montero Días, Selene Núñez Gaspar, Alvaro Santaella Gomez.

**H. U. del Sureste. Arganda del Rey**

Jon Cabrejas Ugartondo, Ana Belén Mancebo Plaza, Arturo Noguerado Asensio, Bethania Pérez Alves, Natalia Vicente López.

**H. de Pozoblanco. Pozoblanco**

José Nicolás Alcalá Pedrajas, Antonia Márquez García, Inés Vargas.

**H. Virgen de los Lirios. Alcoy (Alicante)**

Mª José Esteban Giner.

**Hospital Doctor José Molina Orosa. Arrecife (Lanzarote)**

Virginia Herrero García, Berta Román Bernal.

**H. Nuestra Señora de Sonsoles. Ávila**

Alaaeldeen Abdelhady Kishta.

**C. H. U. de Badajoz. Badajoz**

Rafael Aragon Lara, Inmaculada Cimadevilla Fernandez, Juan Carlos Cira García, Gema Maria García García, Julia Gonzalez Granados, Beatriz Guerrero Sánchez, Francisco Javier Monreal Periáñez, Maria Josefa Pascual Perez.

**H. G. U. de Elda. Elda**

Carmen Cortés Saavedra, Jennifer Fernández Gómez, Borja González López, María Soledad Hernández Garrido, Ana Isabel López Amorós, Maria de los Reyes Pascual Pérez, Andrea Torregrosa García.

**H. U. Puerta del Mar. Cádiz**

José Antonio Girón González, Susana Fabiola Pascual Perez, Cristina Rodríguez Fernández-Viagas, Maria José Soto Cardenas.

**Hospìtal de Montilla. Montilla**

Ana Cristina Delgado Zamorano, Beatriz Gómez Marín, Adrián Montaño Martínez, Jose Luis Zambrana García.

**H. Infanta Elena. Huelva**

María Gloria Rojano Rivero.

**H. U. Quironsalud Madrid. Pozuelo de Alarcón (Madrid)**

Pablo Guisado Vasco, Ana Roda Santacruz, Ana Valverde Muñoz.

**H. de la Axarquía. Vélez-Málaga**

Antonio Lopez Ruiz.

**H. Virgen del Mar. Madrid**

Thamar Capel Astrua, Paola Tatiana Garcia Giraldo, Maria Jesus Gonzalez Juarez, Victoria Marquez Fernandez, Ada Viviana Romero Echevarry.

**Hospital do Salnes. Vilagarcía de Arousa**

Vanesa Alende Castro, Ana María Baz Lomba, Ruth Brea Aparicio, Marta Fernandez Morales, Jesus Manuel Fernandez Villar, Maria Teresa Lopez Monteagudo, Cristina Pérez García, Lorena María Rodríguez Ferreira, Maria Begoña Valle Feijoo.
